# Facing the COVID-19 Pandemic: The Role of Sense of Coherence

**DOI:** 10.3389/fpsyg.2020.578440

**Published:** 2020-11-06

**Authors:** Daniela Barni, Francesca Danioni, Elena Canzi, Laura Ferrari, Sonia Ranieri, Margherita Lanz, Raffaella Iafrate, Camillo Regalia, Rosa Rosnati

**Affiliations:** ^1^Department of Human and Social Sciences, University of Bergamo, Bergamo, Italy; ^2^Family Studies and Research University Centre, Catholic University of the Sacred Heart, Milano, Italy; ^3^Department of Psychology, Catholic University of the Sacred Heart, Milano, Italy

**Keywords:** sense of coherence, COVID-19, well-being, illness, fear

## Abstract

The worldwide outbreak of COVID-19, the ensuing pandemic, and the related containment measures pose considerable challenges to psychological resilience and well-being. Researchers are now forced to look for resources to cope with negative experiences linked to this health emergency. According to the salutogenic approach proposed by Antonovsky, the sense of coherence (SOC) is a major source of resilience. Thus, this study aimed at assessing the role of SOC in moderating the link between illness experiences (in terms of knowing persons diagnosed with COVID-19 and fear of contracting COVID-19) and psychological well-being. 2,784 participants, taken from a large sample of the Italian population (65.4% females) and aged between 18 and 85 years, filled in an anonymous online survey during the 3rd week of the lockdown. Findings supported the moderating role of SOC in shaping the link between illness experiences and psychological well-being. Specifically, participants who knew at least one person diagnosed with COVID-19 showed lower levels of psychological well-being at low levels of SOC. The negative relation between participants’ fear of contracting COVID-19 and psychological well-being was stronger for those who showed higher levels of SOC. This study discusses the implications of these results for interventions aimed at reducing the pandemic’s detrimental effects and promoting resilience.

## Introduction

With the worldwide outbreak of COVID-19, the ensuing pandemic, and the related containment measures, a growing body of research has brought to light the sharp increase in virus-related fears and worries (e.g., Asmundson and Taylor, 2020), mental health problems (see, for reviews, Rajkumar, 2020; Vindegaard and Eriksen Benros, 2020), and social and economic stresses (e.g., Buheji et al., 2020). The COVID-19 crisis left routine coping mechanisms overwhelmed and resulted in feelings of helplessness, lack of control, and loss. One major concern for people is that their acquaintances and relatives or they themselves could get sick by contracting COVID-19 (Pakpour and Griffiths, 2020). This situation poses a considerable challenge to the health system (Vagni et al., 2020) and to psychological resilience (Wang et al., 2020), forcing researchers to identify the resources useful to cope with negative experiences, thoughts, and feelings linked to pandemic and to what has been defined as “parallel pandemic” of acute traumatic stress disorder and of post-traumatic stress disorder, when the stressors and symptoms persist (Mucci et al., 2020).

According to the well-known salutogenic approach of health promotion (Antonovsky, 1979, 1987), a major individual resilience resource is the sense of coherence (SOC). It refers to the global and enduring orientation to view life and the world as “making sense cognitively, instrumentally, and emotionally” (Antonovsky, 1996, p. 15). It is composed of interrelated components: comprehensibility (i.e., the extent to which individuals perceive events as structured, consistent, and clear), manageability (i.e., the extent to which individuals believe that their external or internal resources are adequate to face stressful events), and meaningfulness (i.e., the extent to which individuals perceive life as worthy of commitment and engagement). That is, individuals with a high SOC are likely to perceive stressors as explicable, have confidence in their coping abilities, and feel engaged and motivated to cope with stressors.

Over the years, an impressive amount of psychosocial research provided evidence that people with a strong SOC are less vulnerable to stressful situations. SOC was consistently found to be positively related to health in terms of physical and psychological well-being, self-esteem, self-efficacy, health behaviors, family relationships quality across life adversities, life development span, and cultures (see, for reviews, Flensborg-Madsen et al., 2005; Erikson and Lindström, 2006; Länsimies et al., 2017). SOC does not represent a specific style of coping, but rather helps in choosing the appropriate coping strategy in different kinds of stressful situations (Einav and Margalit, 2020), both acute stress situations (i.e., unexpected facts which overwhelm our resources) and chronic stress situations (i.e., stressors which characterize our life daily). Indeed, all these situations challenge the most routine coping strategies (Paton et al., 2003).

Some studies specifically focused on SOC in highly demanding situations and emergency contexts, such as intergroup conflicts and wars (e.g., Sagy and Braun-Lewensohn, 2009; Kimhi et al., 2010; Veronese et al., 2012; Braun-Lewensohn et al., 2013, 2014), and natural disasters (e.g., Kaiser et al., 1996; Zwiebach et al., 2010; Braun-Lewensohn and Sagy, 2011; Sattler, 2017). For example, the study of Braun-Lewensohn and Sagy (2011), which involved three groups of Israeli adolescents from different cultures (Jews, Muslims, and Druze) in an acute state of stress immediately after a serious bush fire, reported significant negative relationships between SOC and stress reactions (i.e., state anxiety, state anger, and psychological distress). In their study with adolescents before and after disengagement from the Gaza Strip, Braun-Lewensohn et al. (2013) found that SOC weakened immediately after the disengagement, but remained the main protective factor against the stress reactions (i.e., anxiety and anger) a few months post-disengagement. Similarly, Kaiser et al. (1996) found that SOC was negatively associated with psychological distress, depression, and anxiety in a sample of young-adults and adults 1 month following Hurricane Hugo. From the recent meta-analysis of Schäfer et al. (2019) on the heterogenous literature which investigated the link between SOC and post-traumatic stress disorder symptoms’ severity, it emerged a substantial negative link between these two variables. Participants with higher SOC levels showed lower symptom severity. Moreover, high-SOC individuals recovered more rapidly and even experienced post-traumatic growth (e.g., new possibilities, relating to others, and appreciation of life). The authors concluded that, in the aftermath of a traumatic event, SOC can provide individuals with confidence in their ability to cope with the adversity by using the so-called general resistance resources^1^ and the strength to resume their prior assumptions of a comprehensible and meaningful world.

A few recent studies focused on SOC as the mechanism underpinning the stress-health link by analyzing it as a moderator. However, while SOC has consistently shown positive direct associations with health, its moderating role in the stress-health link needs further clarification (Richardson and Ratner, 2005; Mc Gee et al., 2018). Indeed, the ability of SOC to buffer the negative effects of stressful experiences on health might depend on the type and severity of stressful events and on the health indicators considered (see, for example, Quehenberger and Krajic, 2017).

From all the above considerations, it is evident that SOC may be a powerful protective factor to reduce stress imposed by the virus outbreak and promote well-being. In their inspiring work (reporting results from a panel study carried out in Germany), Schäfer et al. (2020) pointed out that SOC predicted changes in psychopathological symptoms from COVID-19 pre-outbreak (at the end of February) to post-outbreak (1 month later). Results showed that a significant proportion of the sample experienced mental health problems related to the COVID-19 pandemic (especially among women and younger participants), but higher pre-outbreak levels of SOC were related to smaller clinically relevant changes in psychopathology (i.e., increases or decreases). That is, higher levels of SOC buffered the impact of COVID-19 stressors on general health, but did not result systematically in lower symptom levels.

## The Present Study

To our knowledge, research of Schäfer et al.’s (2020) is the only one that empirically considers SOC in relation to COVID-19. Thus, our general aim was to deepen the understanding of the role of SOC in psychological reactions to the pandemic. We were interested in analyzing whether and the extent to which SOC moderates the relation between COVID-19 illness experiences (in terms of knowing people diagnosed with COVID-19 and fear of contracting COVID-19) and psychological well-being. We expected respondent’s well-being to be negatively related to knowing people diagnosed with COVID-19 and higher levels of fear of contracting COVID-19 (H1) and to be positively related to higher levels of SOC (H2). We moreover expected SOC to weaken the negative effects of illness experiences on well-being (H3). In testing these hypotheses, we involved a large sample from the Italian population. As known, Italy has been severely hit by the COVID-19 pandemic with one of the highest number of infections and deaths (Italian Health Minister, http://www.salute.gov.it/portale/home.html). Thus, it is likely that Italian people have lived for a prolonged period of time under highly stressful conditions, making studies on SOC (as well as on other resistance resources; see, Antonovsky, 1979, 1987) particularly relevant. Moreover, Italy is characterized by a socio-cultural context and health system completely different from those of East Asian countries where most of the COVID-19 studies have been carried out so far. Research with this population is urgently needed for the development of more culturally appropriate interventions to manage the psychological consequences of the COVID-19 pandemic (Giallonardo et al., 2020).

## Materials and Methods

### Participants

This study was part of a wider research “The Family at the time of COVID-19” carried out by the Family Studies and Research University Centre of the Università Cattolica del Sacro Cuore which included a large sample of the Italian population. In the current study we considered 2,784 participants (males = 34.6%, *N* = 964; females = 65.4%, *N* = 1820) aged between 18 and 85 years who responded to all questions of interest; 54.8% of them were aged below 45 years and 45.2% above 45 years. With regard to the place of residence, 45% of the participants were from the North of Italy, 19% from Central Italy, and 36% lived in the South of Italy or on an island. Regarding the level of education, 10.3% had completed primary school, 54.3% had completed high school, and 35.4% had a university degree. The study was approved by the Ethics Committee of the Department of Psychology of the Università Cattolica del Sacro Cuore (protocol number 15–20) and it followed the APA ethical guidelines for human research.^2^ All participants provided an electronic informed consent prior to their participation; having at least 18 years was the only inclusion criterion adopted. The enrolled participants were asked to complete an anonymous online survey which was broadcasted through different platforms and mainstream social-media with the collaboration of Human Highway Society. The questionnaire was administered between March 30 and April 7, 2020, during the 3rd week of the lockdown imposed by the Italian Prime Minister on March 11, 2020.

### Measures

The questionnaire included questions on demographic information and the following measures.

#### Knowing People Diagnosed With COVID-19

Participants were asked to answer the following question: “Do you know someone who got sick because of COVID-19?” (0 = no, 1 = yes).

#### Fear of Contracting COVID-19

Participants were asked to answer the following question: “Are you afraid of getting sick because of COVID-19?” (from 1 = not at all to 7 = a lot).

#### Sense of Coherence

The Italian version of the *Sense of Coherence Scale* (Antonovsky, 1987; Barni and Tagliabue, 2005; see Holmefur et al., 2015 for the full scale^3^), composed by 11 items (from 1 = very seldom or never, to 7 = very often), was used to measure the individual level of SOC during the COVID-19 pandemic. Item examples are “I have feelings I’m in an unfamiliar situation and I don’t know what to do” and “I have feelings I’m not sure I can keep under control.” The exploratory factor analysis (Principal Component extraction and Varimax rotation) supports the one-factor solution with 43.26% of variance explained and satisfactory communalities (mostly above 0.50). The total score was obtained by averaging the scores of the 11 items. Higher scores indicated higher levels of SOC. Cronbach’s alpha to assess the internal consistency of the scale was 0.86.

#### Psychological Well-Being

Based on our study aims, four items of the *Mental Component Summary* of the *Short-Form Health Survey* (SF-12; Italian version by Apolone and Mosconi, 1998; Apolone et al., 2001) were selected, measuring an individual’s overall psychological well-being in terms of vitality (having a lot of energy), mental health (feeling calm and peaceful), and social functioning (interference of physical health or emotional problems with social activities). An item example is “I felt full of energy.” Participants reported about their well-being during the preceding week. Raw scores for items ranged from 1 (never) to 6 (always). The total score was considered for the current study and was computed by averaging the scores of the four items; a higher score indicated a higher level of psychological well-being. Cronbach’s alpha of the scale was 0.75.

### Data Analysis

Initially, we described the study variables in terms of means, ranges, and SDs. After calculating bivariate Pearson correlations among variables, we carried out a hierarchical regression model to test the moderation hypothesis. We first controlled for respondents’ gender, age, and geographical area (Step 1). Knowing people diagnosed with COVID-19, fear of contracting COVID-19, SOC (Step 2), and their interactions (Step 3) were the predictors, while well-being was the criterion variable. The continuous predictors were centered on their means before computing the interaction terms to minimize multicollinearity and for easier interpretation of model coefficients (Aiken and West, 1991). Lastly, simple slope analysis was performed to probe any significant interaction effect. We used SPSS 24.0 to conduct all the analyses.

## Results

Table 1 shows means, SDs, and ranges of the study variables as well as the Pearson correlations among them.

**Table 1:** Means, SDs, ranges, and correlations among the study variables.

	Mean (SD)	Range	1.	2.	3.	4.
Knowing people diagnosed with COVID-19	-	-	1			
Fear of contracting COVID-19	4.59 (1.73)	1.00–7.00	0.07^***^	1		
Sense of coherence	4.69 (1.17)	1.00–7.00	−0.02	−0.20^***^	1	
Psychological well-being	3.59 (0.91)	1.00–6.00	−0.07^***^	−0.27^***^	0.59^***^	1

Knowing people diagnosed with COVID-19: 0 = no, 1 = yes.

*** *p* < 0.001.

Descriptive analyses also showed that 27% of the participants declared to know at least one person diagnosed with COVID-19 and 73% did not. Table 2 presents the moderation analyses results.^4^

**Table 2:** Moderation analysis results (criterion variable: psychological well-being).

Predictor	b	ß	95% CI	*R*^2^
Step 1 (*R*^2^ = 0.049)				
Gender	−0.39^***^	−0.20^***^	(−0.457, −0.316)	0.043
Age	0.06	0.04	(−0.00, 0.133)	0.002
Center	−0.08	−0.03	(−0.175, 0.010)	0.000
South and Islands	−0.14^***^	−0.07^***^	(−0.212, −0.061)	0.004
Step 2 (*R*^2^ = 0.401)				
Knowing people diagnosed with COVID-19	−0.13^***^	−0.07^***^	(−0.197, −0.074)	0.010
Fear of contracting COVID-19	−0.07^***^	−0.14^***^	(−0.088, −0.056)	0.054
Sense of coherence	0.43^***^	0.55^***^	(0.405, 0.452)	0.288
Step 3 (*R*^2^ = 0.406)				
Knowing people diagnosed with COVID-19^*^ sense of coherence	0.07^*^	0.04^*^	(0.014, 0.122)	0.002
Fear of contracting COVID-19^*^ sense of coherence	−0.02^***^	−0.06^***^	(−0.037, −0.012)	0.003

Gender: 0 = male, 1 = female. Age: 0 = up to 44 years, 1 = 45 years and above. Knowing people diagnosed with COVID-19: 0 = no, 1 = yes. CI = confidence interval for estimate.

* *p* < 0.05;

*** *p* < 0.001.

The regression model showed that a significant portion of variance in participants’ well-being was explained by the predictors (i.e., knowing people diagnosed with COVID-19, fear of contracting COVID-19, SOC) and their interactions, with *R*^2^ = 0.406. The analysis also revealed the existence of gender and geographical area differences on psychological well-being (*R*^2^ = 0.049). Specifically, women and people from Southern Italy and islands reported lower levels of well-being.

**Figure 1:**
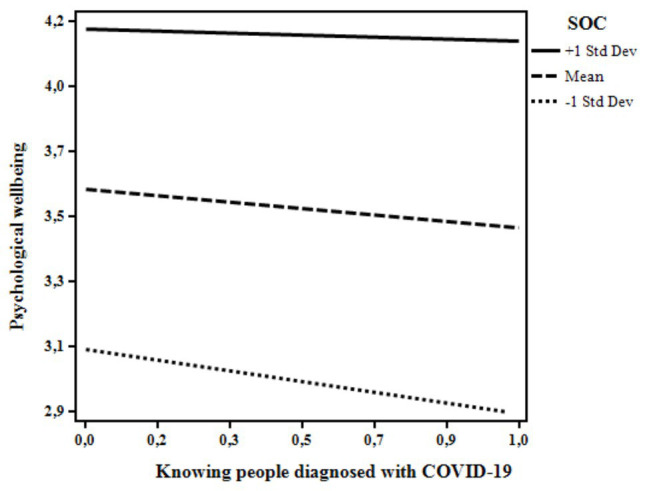
The moderating role of sense of coherence (SOC) on the relation between knowing people diagnosed with COVID-19 and psychological well-being. Knowing people diagnosed with COVID-19: 0 = no, 1 = yes.

**Figure 2:**
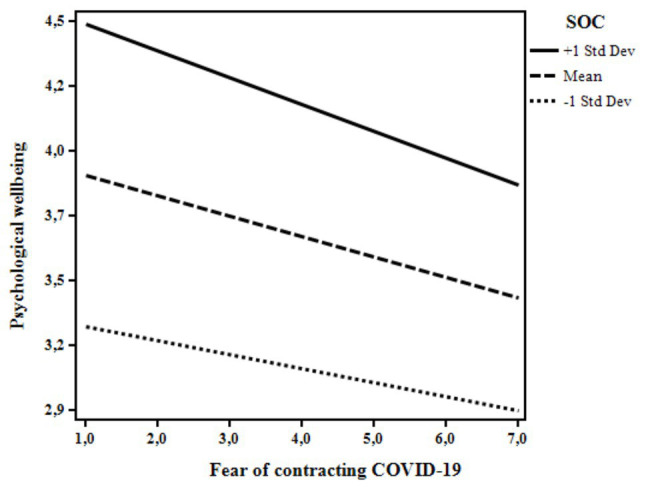
The moderating role of SOC on the relation between fear of contracting COVID-19 and psychological well-being.

From the inspection of the β weights it was possible to see that the more the participants reported to know people diagnosed with COVID-19 and fear of contracting COVID-19, the lower their psychological well-being. In contrast, SOC positively related to the participants’ well-being. Interestingly, two statistically significant moderations emerged from the analyses. First, when knowing at least one person diagnosed with COVID-19, lower levels of well-being were revealed for those with low levels of SOC. At high levels of SOC, no differences in well-being were evident between those who did or did not know someone diagnosed with COVID-19 (Figure 1). Secondly, the negative relation between the participants’ fear of contracting COVID-19 and their psychological well-being was slightly stronger for those who showed higher levels of SOC (Figure 2).

## Discussion

This study extended the research on SOC in highly stressful situations, assessing its role in supporting psychological well-being in face of the COVID-19 pandemic. Although the direct positive link between SOC and health is largely documented in literature (e.g., Flensborg-Madsen et al., 2005; Erikson and Lindström, 2006; Länsimies et al., 2017), the moderating role of SOC in the stress-health relation is still not clear (e.g., Richardson and Ratner, 2005; Mc Gee et al., 2018). To our knowledge, this is the first study to analyze whether and the extent to which SOC moderates the relationship between COVID-19 illness experiences (in terms of knowing people diagnosed with COVID-19 and the fearing to contract COVID-19) and psychological well-being on a large sample of Italian individuals.

First, results showed that women and people from Southern Italy and islands reported lower levels of psychological well-being. Generally speaking, women’s psychological well-being tends to be lower compared to their male counterparts (e.g., Lim et al., 2018), and our findings confirmed that gender affects individuals’ mental health in the same direction also when related to the COVID-19 pandemic (Mazza et al., 2020; Wang et al., 2020). With regards to people from Southern Italy and islands, despite being less affected by the COVID-19 diffusion compared to Northern Italy, they expected the arrival of the virus there as well, where the health system would have faced great difficulty (Paterlini, 2020). This may have negatively affected their well-being.

In line with a study dealing with psychological distress among Italian people during the COVID-19 pandemic (Mazza et al., 2020) and with our first hypothesis (H1), the more the participants reported knowing people diagnosed with COVID-19 and also feared getting sick themselves, the lower was their psychological well-being. Moreover, according to the literature (e.g., Braun-Lewensohn et al., 2013; Sattler, 2017) and to our second hypothesis (H2), SOC was positively associated with psychological well-being, confirming its critical role in helping individuals cope with stressors and traumatic experiences also in the context of the COVID-19 pandemic.

The moderation models tested in this study confirmed the buffering role of SOC in moderating the link between the illness experiences and psychological well-being, also controlling for participants’ gender, age, and geographical area. The co-occurrence of knowing someone who got sick and the low level of SOC was associated with lower levels of psychological well-being, this partially confirming out third hypothesis (H3). People who are in “close contact” with COVID-19 may be particularly overwhelmed especially if they feel that they have a low sense of control over the situation. This result made us consider that knowing someone who got sick is an experience that can be rationally realized and managed. In these situations, a clear and consistent perception of the events and the possibility to adequately face them may represent a crucial resource, buffering the detrimental association between the “close contact” with COVID-19 and psychological well-being. More interestingly, and unlike our third hypothesis (H3), fear of contracting COVID-19 was slightly more negatively associated with psychological well-being for individuals with higher levels of SOC. In interpreting these findings, we should consider that fear of getting sick is an emotional reaction that may even be considered adaptive, thus serving to mobilize energy to deal with stressful situations and adopt protective measures. Indeed, research has largely documented that worries regarding physical diseases and risk perception are strictly interrelated predictors of health behaviors (e.g., Kwak et al., 2009; Park et al., 2009; Shiloh et al., 2013). In the specific situation of the COVID-19 pandemic, fear of getting sick associated to high levels of SOC could lead to a lower psychological well-being as seen in this study because of the unpredictability of the situation. Indeed, Italy has been severely hit by the pandemic and it was the second country after China where the lockdown was imposed to the population. Data was collected only 3 weeks after the beginning of the lockdown, namely a moment of deep acute stress, and no clear examples and expectations of how the situation would have turned out were available. Therefore, the fear may have caused a worse scenario for those who were instead more likely to see the world as “making sense cognitively, instrumentally, and emotionally” (Antonovsky, 1996, p. 15). However, we may speculate that fear may even promote healthy behaviors as occurred in similar contexts (e.g., practicing social distancing, hand hygiene, properly using face masks). Further research should try to corroborate this finding.

This study has some limitations. Firstly, the study used a cross-sectional design. Hence, we could not examine the bidirectionality of the emergent links and draw casual inferences from the results. Besides, a longitudinal approach may be useful to explore also the relatively long-lasting exposure to stressors related to the COVID-19 and the long-term impact of this crisis. Secondly, potential confounding variables (e.g., whether the COVID-19 affected person was a relative, a very close person, or simply an acquaintance, and the severity of their illness) in the relationships between the study variables should be considered when interpreting our results and should be included in future studies. Thirdly, due to the nature of the phenomena investigated, we could not rely on validated measures to assess COVID-19 illness experiences and only *ad hoc* items were used. Furthermore, because of the COVID-19 outbreak, an online survey was administered, excluding those who do not use network devices. We can speculate that we have excluded part of the population who is not likely to use platforms and mainstream social-media to fill in online questionnaires.

Despite these limitations, the main findings of this study offer some practical implications for interventions aimed at reducing pandemic detrimental effects and promoting resilience. According to the “3*C*s” (Control, Coherence, and Connectedness) model developed by Reich (2006) to account for resilience resources in emergency situations, it seems relevant to support individuals in perceiving critical events as clear and explicable and in developing a sense of confidence in their coping abilities. Indeed, Sethuraman (2020) suggests practical strategies to medical professionals to foster SOC (e.g., promote comprehension of evidence-based scientific information and provide manageable options to cope with the pandemic, or make it meaningful to the people) among counsel patients. The utility of promoting SOC seems to apply also to the general population; in particular it becomes useful to combine the promotion of the ability of making sense of the experiences, even the most stressful ones, with the ability in coping with emotional reactions of fear. However, it is important to be aware that this may vary according to different experiences of illness considered.

## Data Availability Statement

The raw data supporting the conclusions of this article will be made available by the authors, without undue reservation.

## Ethics Statement

The studies involving human participants were reviewed and approved by Ethics Committee of the Department of Psychology, Università Cattolica del Sacro Cuore, Milano, Italy. The patients/participants provided their written informed consent to participate in this study.

## Author Contributions

DB and FD contributed to the data analysis and wrote the manuscript. EC, LF, and SR contributed to the writing of the manuscript. ML, RI, and CR designed and carried out the study. RR designed and carried out the study and contributed to the writing of the manuscript. All authors contributed to the article and approved the submitted version.

### Conflict of Interest

The authors declare that the research was conducted in the absence of any commercial or financial relationships that could be construed as a potential conflict of interest.

## Footnotes

^1^The term generalized resistance resources (e.g., material resources, knowledge and intelligence, social support, etc.) includes all the characteristics of a person, a group or a community that facilitate the individual’s abilities to effectively cope with stressors and contribute to the development of the individual’s level of SOC (Antonovsky, 1979,^1987^).

^2^http://www.apa.org/ethics/code/

^3^In the Italian validation of the scale by Barni and Tagliabue (2005) two items (“Has it happened in the past that you were surprised by the behavior of people whom you thought you knew well?” and “Has it happened that people whom you counted on disappointed you?”) were eliminated to reach an improved and completely acceptable model.

^4^We also checked for statistically significant differences with regard to the COVID-19 illness experiences depending on respondents’ gender, age, and geographical area. Participants from Northern Italy declared to a higher extent to know someone who got sick because of COVID-19 (16.3%) compared to others (Center: 4.5%; South: 6.2%; χ2(2) = 105.53, *p* < 0.01). Women (M = 4.77, SD = 1.72) and respondents from the South and islands (M = 4.72, SD = 1.77) showed a higher fear of getting sick because of COVID-19 (men: M = 4.27, SD = 1.71; *F*(1, 2674) = 50.77, *p* < 0.01; North: M = 4.53, SD =1.72 and Center: M = 4.49, SD = 1.68; *F*(2, 2655) = 4.26, *p* < 0.05).
